# Comparing DNA methylation profiles across different tissues associated with the diagnosis of pediatric asthma

**DOI:** 10.1038/s41598-019-56310-4

**Published:** 2020-01-13

**Authors:** Ping-I Lin, Huan Shu, Tesfaye B. Mersha

**Affiliations:** 1 Department of Health Sciences, Karlstad University, Karlstad, Sweden; 2 Department of Environmental Science and Analytical Chemistry, Stockholm University, Stockholm, Sweden; 3 Division of Asthma Research, Department of Pediatrics, Cincinnati Children’s Hospital Medical Center, University of Cincinnati, Cincinnati, OH USA

**Keywords:** Asthma, Risk factors

## Abstract

DNA methylation (DNAm) profiles in central airway epithelial cells (AECs) may play a key role in pathological processes in asthma. The goal of the current study is to compare the diagnostic performance of DNAm markers across three tissues: AECs, nasal epithelial cells (NECs), and peripheral blood mononuclear cells (PBMCs). Additionally, we focused on the results using the machine learning algorithm in the context of multi-locus effects to evaluate the diagnostic performance of the optimal subset of CpG sites. We obtained 74 subjects with asthma and 41 controls from AECs, 15 subjects with asthma and 14 controls from NECs, 697 subjects with asthma and 97 controls from PBMCs. Epigenome-wide DNA methylation levels in AECs, NECs and PBMCs were measured using the Infinium Human Methylation 450 K BeadChip. Overlap analysis across the three different sample sources at the locus and pathway levels were studied to investigate shared or unique pathophysiological processes of asthma across tissues. Using the top 100 asthma-associated methylation markers as classifiers from each dataset, we found that both AEC- and NEC-based DNAm signatures exerted a lower classification error than the PBMC-based DNAm markers (p-value = 0.0002). The area-under-the-curve (AUC) analysis based on out-of-bag errors using the random forest classification algorithm revealed that PBMC-, NEC-, and AEC-based methylation data yielded 31 loci (AUC: 0.87), 8 loci (AUC: 0.99), and 4 loci (AUC: 0.97) from each optimal subset of tissue-specific markers, respectively. We also discovered the locus-locus interaction of DNAm levels of the CDH6 gene and RAPGEF3 gene might interact with each other to jointly predict the risk of asthma – which suggests the pivotal role of cell-cell junction in the pathological changes of asthma. Both AECs and NECs might provide better diagnostic accuracy and efficacy levels than PBMCs. Further research is warranted to evaluate how these tissue-specific DNAm markers classify and predict asthma risk.

## Introduction

Asthma affects 11 million children in the U.S., and has been on the rise over the past two decades^1^. Although the heritability estimate may reach 80%^2^, the rapid increment in its incidence cannot be simply explained by genetic factors alone. Epigenetics may account for the mechanisms underlying environmental effects on genetic functions^3^, and hence may play a role in the environment-related pathogenesis of asthma^4^. DNA methylation (DNAm) is the first identified epigenetic mechanism that been extensively studied in allergic disease^5–8^. Emerging evidence has suggested that DNAm can be considered as a robust epigenetic marker that could aid clinical applications, such as diagnostics^9^.

One of the major challenges of DNAm research on asthma is the selection of the target tissue for DNA samples. The direct access to the lung tissue DNA which is primarily involved in asthma pathophysiology is limited^10^. Surrogate markers from easily accessible tissues are the markers of choice for studies involving large-scale screening and children^11,12^. Most asthma epigenetic studies have relied on easily accessible surrogate tissues such as peripheral blood mononuclear cells (PBMCs)^13–15^. However, DNAm tend to be highly tissue- or cell-type specific^16–20^, so PBMCs-based methylation patterns might not be correlated with airway epithelial cell-based (AEC-based) methylation patterns^21^. Another surrogate tissue is nasal epithelial cells (NECs). Accumulating evidence has suggested that NECs are biologically relevant proxies for AECs^22–24^. NECs can also be readily and easily sampled during asthma attacks, which can help to understand the pathophysiological changes in bronchial airways^25,26^ It remains unclear whether DNAm signatures from peripheral tissues, such as PBMCs or NECs can provide better surrogate markers to facilitate the diagnostics of asthma. In addition, little is known regarding the relationship among DNAm profiles of PBMCs, NECs, and AECs in asthma.

Furthermore, most previous studies have implemented the single-locus model to identify the trait-associated loci with variable DNAm levels – which might have ignored the joint effects of epigenetic modifications over multiple loci. Therefore, the overarching goal of this study is to systemically compare the diagnostic performance of different tissue-specific DNAm datasets. In addition, we have evaluated the diagnostic (or prediction) performance of these tissue-specific DNAm markers, in both single-locus and multi-locus models. We anticipated that these results would clarify which type of surrogate marker might yield a better diagnostic accuracy and help to gain some insight into tissue-specific DNAm patterns underlying the development of asthma.

## Materials and Methods

### Methylation data collection

The data used in this study was retrieved from the publicly available Gene Expression Omnibus (GEO) database at NCBI (http://www.ncbi.nlm.nih.gov/geo/)^27,28^. We obtained DNAm data from airway epithelial cells (AECs), nasal epithelial cells (NECs), peripheral blood mononuclear cells (PBMCs), respectively^29–31^. Each individual dataset was uploaded to GEO by the original study groups^28,32^. The information of (1) GEO accession, (2) sample type, (3) platform, (4) numbers of asthmatic and control individuals, can be found in Table 1. Visual BASIC macros were used to extract the expression values of individual genes in each sample. A total of 485,000 probes covering 25,000 genes were taken for the analysis.

**Table 1:** DNA methylation datasets used for the present study. NCBI GEO (Gene Expression Omnibus) accession number, tissue/cell sample type, DNA methylation platform, and sample size have been shown for each study.

Data Type	GEO ID	Sample type*	Platform	Sample size (cases/controls)	References
Primary tissue	GSE85568	AECs	Infinium Methylation 450k BeadChip	74/41	^30^
Surrogate tissue	GSE109446	NECs	Infinium Methylation 450k BeadChip	15/14	^29^
	GSE40736	PBMCs	Infinium Methylation 450k BeadChip	97/97	^31^

^*^AECs = airway epithelial cells, NECS = nasal epithelial cells, PBMCs = peripheral blood mononuclear cells.

### DNA methylation data processing

The epigenome-wide DNA methylation scan was conducted in three datasets: AECs (74 cases and 41 controls), NECs (15 cases and 14 controls), and PBMCs (97 cases and 97 controls), using the Infinium HumanMethylation450 BeadChip. We constructed data tables containing DNA methylation values using GEO2R, a web based limma R packages from the Bioconductor project. GEOquery R package was used to parse GEO data into R data structures that can be used by other R packages. The original datasets deposited to the Gene Expression Omnibus database have gone through QC processes described elsewhere^33^. Briefly, probes located on the sex chromosomes and those that had a detection P value of greater than 0.01 in 75% of samples were removed. Probes mapped to more than one location in a bisulfite-converted genome or overlapped with the location of known SNPs were also excluded. Infinium type I and type II probe biases were corrected for using the SWAN algorithm. Therefore, we believe that the biases due to cross-hybridization have been minimized.

### Statistical analysis

#### Genome-wide methylation association analysis

Data from the methylation array were normalized with the Subset-quantile Within Array Normalization (SWAN) method contained in the R package minfi^34^, and the normalized M-values were used in all downstream analyses. Normalized data was used to run the generalized linear model that adjusted for age, sex, and the surrogate variable detected by R function “sva” (to reduce unwanted bias caused by batch effects). The analyses were performed in samples from PBMCs, NECs, and AECs, independently. We also used heat maps to visualize how the top 100 asthma-associated loci which were ranked by the p-value, from each dataset could distinguish cases from controls using the clustering analysis with Euclidean distance metric and Ward’s minimum variance method as the linkage algorithm. The classification errors were then calculated in each cluster. As graphical representation and visually assess the quality of each individual dataset, hierarchical cluster analysis was performed using Genesis software^35^.

#### Functional enrichment, pathways and networks

In order to gain further insight into the functional significance, we used two approaches to identify pathways overlap among AECs, NECs, and PBMCs based on enriched candidate genes in each study. The overlapping frequency analysis demonstrates how often the same set of genes is selected from different samples sources and which tissues tend to select the same set of genes. We selected the top 100 asthma-associated loci from each study and then used the webtool at ConsensusPathDB (http://cpdb.molgen.mpg.de/) to perform over-representation analyses^36^. The analysis criteria included: (1) one-next neighbors for the radius with p-value < 0.01, (2) pathway-based sets at least two overlapped genes and p-value < 0.01, and (3) gene ontology level 2 categories with p-value < 0.01. The results from the second approach helped visualize the possible “hub” pathway from the networks associated with the candidate genes.

Concordance rate. The concordance rate measures the proportion of shared genes among the top ranked genes in AECs, NECs, and PBMCs in the tissue-stratified analysis. Genes were ranked using the p-value from the most to least significant, and the top ranked 100 genes were identified in each tissue. If **m** is the number of overlapped genes within the top **t** percentile (t = 1, 2, 3… 100) in AECs, NECs and PBMCs, and **N** is the total number of genes analyzed, then the concordance rate (ζ) is defined as:

$$\zeta =\frac{100m}{tN}$$

Correlation among AECs, NECs and PBMCs. We calculated the correlation in differentially methylated genes among tissues and determine whether readily accessible tissue could be used as a reliable surrogate marker to predict DNA methylation in less accessible tissues, which would facilitate the development of novel differential methylation-based models for assessing asthma risk and progression. We used the Pearson correlation coefficient to compare the fold change differences between AECs, NECs and PBMCs. The Pearson’s correlation coefficient between the fold change X in AECs or NECs and Y in PBMCs is defined as:

$$r=\,\frac{{\sum }_{i}({x}_{i}-\bar{x})({y}_{i}-\bar{y})}{\sqrt{{\sum }_{i}{({x}_{i}-\bar{x})}^{2}{\sum }_{i}{({y}_{i}-\bar{y})}^{2}}},$$

where *x*_*i*_ and *y*_*i*_ are the fold changes of the i^th^ gene in AECs or NECs and PBMCs, respectively; $\bar{x}$ and $\bar{y}$ are the average of X and Y, respectively. The correlation coefficient (*r*) ranges from −1 to 1, with *r* closer to 1 or −1 indicating a monotonically increasing or decreasing relationship and *r* closer to 0 signifying weak or no relationship.

Jaccard Index (J). The Jaccard index was used at the gene and pathway level to determine relatedness between asthma tissues/cells in terms of differentially methylated genes and pathways^37^. We considered the similarity between two tissues as the number of genes (or pathways) shared divided by the total number of genes (or pathways) present in either of them. It is expressed as follows: J = C/(A + B − C), where A is the number of genes present in tissue A; B is the number of genes present in tissue B; and C is the number of genes present in both tissue A and tissue B. The number of genes present in either of the tissues is given by A + B − C. The Jaccard Index ranges from 0 to 1, where a higher value indicates a higher similarity between two tissue groups.

### Prediction accuracy

We used random forest (RF) classification to identify two optimal sets of predictors (i.e., loci with a variation in methylation levels) from each dataset. To identify the optimal set of predictors, we used the R package “AUCRF” to calculate the out-of-bag (OOB) error by implementing the backward elimination based on the initial ranking of importance of the variables. The OOB method has been used in several machine learning models utilizing bootstrap aggregation (bagging) to sub-sample data samples used for training to measure the performance of the predictor^38^. We used “mean decrease accuracy” as the primary parameter for variable-specific importance. The higher this value is, the more important this predictor could be. When the area-under-the-curve based on the OOB errors reached the peak value, we could determine which predictors might constitute the optimal set.

### Identifying the optimal subset of probes to predict the diagnosis

We used the random forest classification (RF) algorithm to rank the top 100 asthma-associated loci. We used the R function “randomForestExplainer” to plot the relationship between two parameter values: “Gini_increase” and “accuracy_decrease.” “Gini_increase” indicates the increase in the Gini impurity index, and “accuracy_decrease” refers to the mean decrease of prediction accuracy after the corresponding predictor was permuted. The Gini impurity index was calculated as $\mathop{\sum }\limits_{t=0}^{t=k}{P}_{t}(1-{P}_{t})$, where k is the number of lasses in the target variable and P_t_ indicates the ratio of this class (i.e., asthma). The higher Gini impurity index means that the CpG site could be a better distinguisher between cases and controls.

### Locus-by-locus interactions

To compare the prediction accuracy in the context of locus-by-locus interactions, we further calculated the conditional minimum depth of trees using the R function “randomForestExplainer.” We selected the top 5 pairs of loci consisted of a root variable and another variable ranked by their conditional minimum depths. The interactions were visualized using the signals that underwent inverse rank-based transformation so the results from different datasets can be compared. The final set of validation analysis with generalized linear model that regresses the outcome against two different CpG methylations and their interaction was performed using the software STATA SE^39^.

## Results

**Figure 1:**
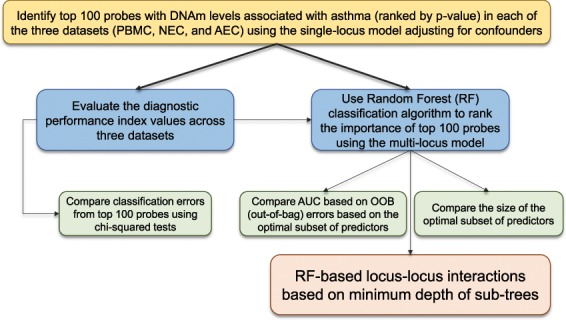
The workflow of the step-wise analysis plans is shown. The framework illustrates how we explored different ways of evaluating the diagnostic performance beyond the one-locus model (using parametric linear model). We proposed to use Random Forest classification algorithm to identify the optimal subset of predictors in the context of multi-locus effects. It also includes how we investigated the relationships among the genes that harbor different tissue-specific asthma-associated DNAm signatures.

Figure 1 illustrates an overview of the experimental workflow to achieve this goal. In Step 1, first we curated three different public data sets and extensively evaluated the differences in DNA methylation profiles between asthmatics and controls. Second, we performed cell/tissue-based DNA methylation analysis. Generalized linear model was used to determine differentially methylated genes within dataset. Third, we selected the top 100 probes from each dataset (i.e., PBMC, NEC, and AEC) in the context of the single-locus EWAS model after controlling for sex, age, and batch effect. Step 2 was to evaluate the diagnostic accuracy based on these top 100 probes from each dataset based on the classification error. Step 3 was to generate the three lists of candidate genes based on each dataset’s top 100 probes, and then perform two sets of analyses. The first set of analysis was using the gene set enrichment analysis to identify the biological pathways, where each dataset’s candidate genes were over-represented. The second set of analysis was to use the correlation test to evaluate the degree of overlaps at both the genetic and pathway levels. Step 4 was to use Random Forest classification algorithm (RF) to rank the relative importance of the probes among the original top 100 probes in the context of a mixture of multiple predictors (i.e., multi-locus model). Step 5 was to assess the predictor error corresponding to the removal of the optimal subset of probes in order to compare the diagnostic performance of this optimal subset of predictors across the three datasets. The size of the optimal subset of probes was used to evaluate the diagnostic efficiency (i.e., a lower number of probes in the optimal subset of predictors might indicate a better diagnostic efficiency). The final step was to identify the potential pair of probes that interact with each other to jointly predict the diagnosis based on the minimum depth of sub-trees from the RF analyses. The statistical significance of the interaction effect was then evaluated using the logistic regression model that regressed the binary outcome against each probe and the product composed of two probes while adjusting for confounders. Finally, we performed tissue functional enrichment analysis to explore the biological insights underpinning asthma pathogenesis.

### EWAS based on the one-locus model

**Figure 2:**
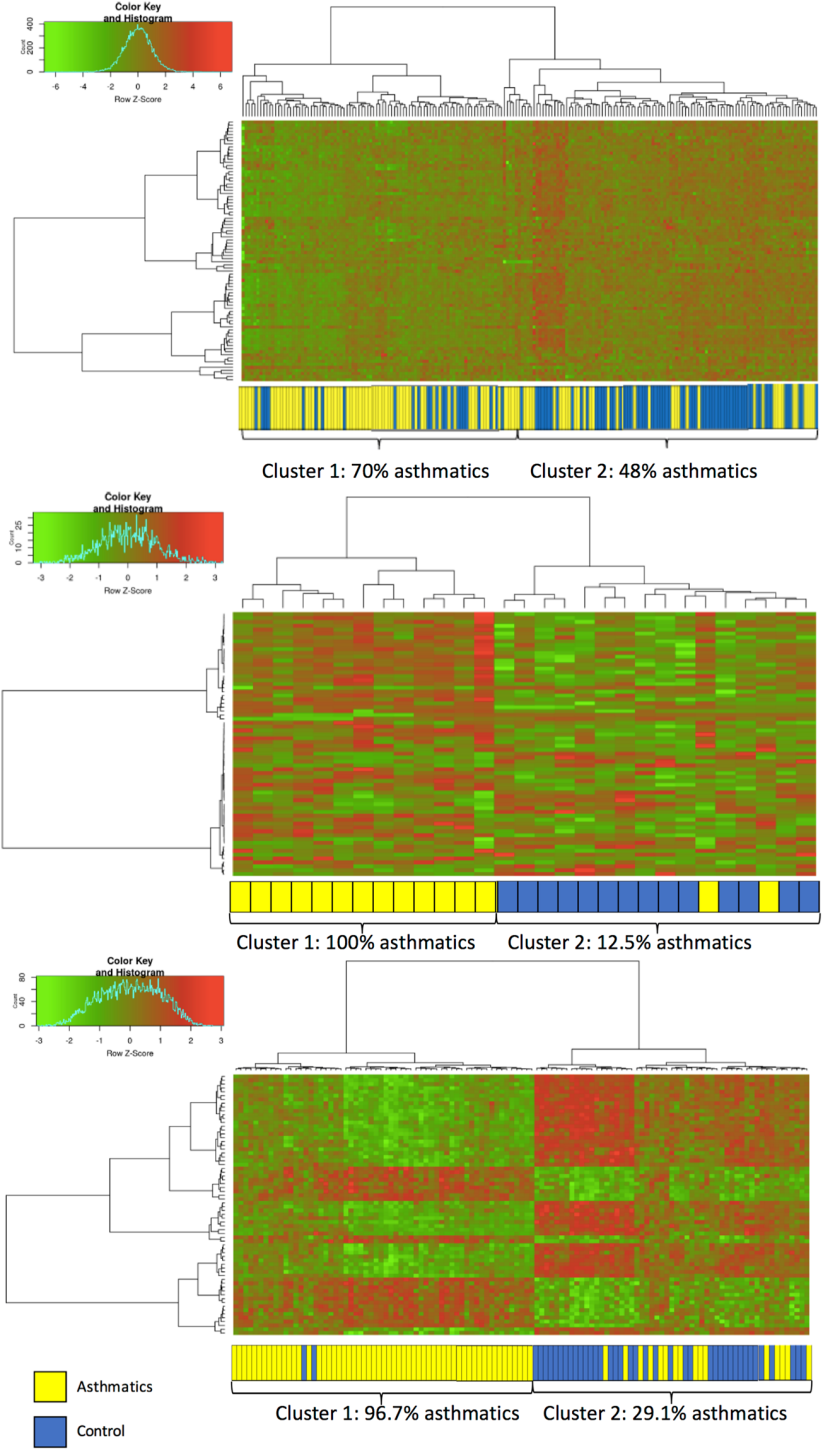
(**A**) The heat maps show the clustering analysis results from significantly asthma-associated methylated loci based on DNA extracted from PBMC. (**B**) The heat maps show the clustering analysis results from significantly asthma-associated methylated loci based on DNA extracted from NEC. (**C**) The heat maps show the clustering analysis results from significantly asthma-associated methylated loci based on DNA extracted from AEC.

**Figure 3:**
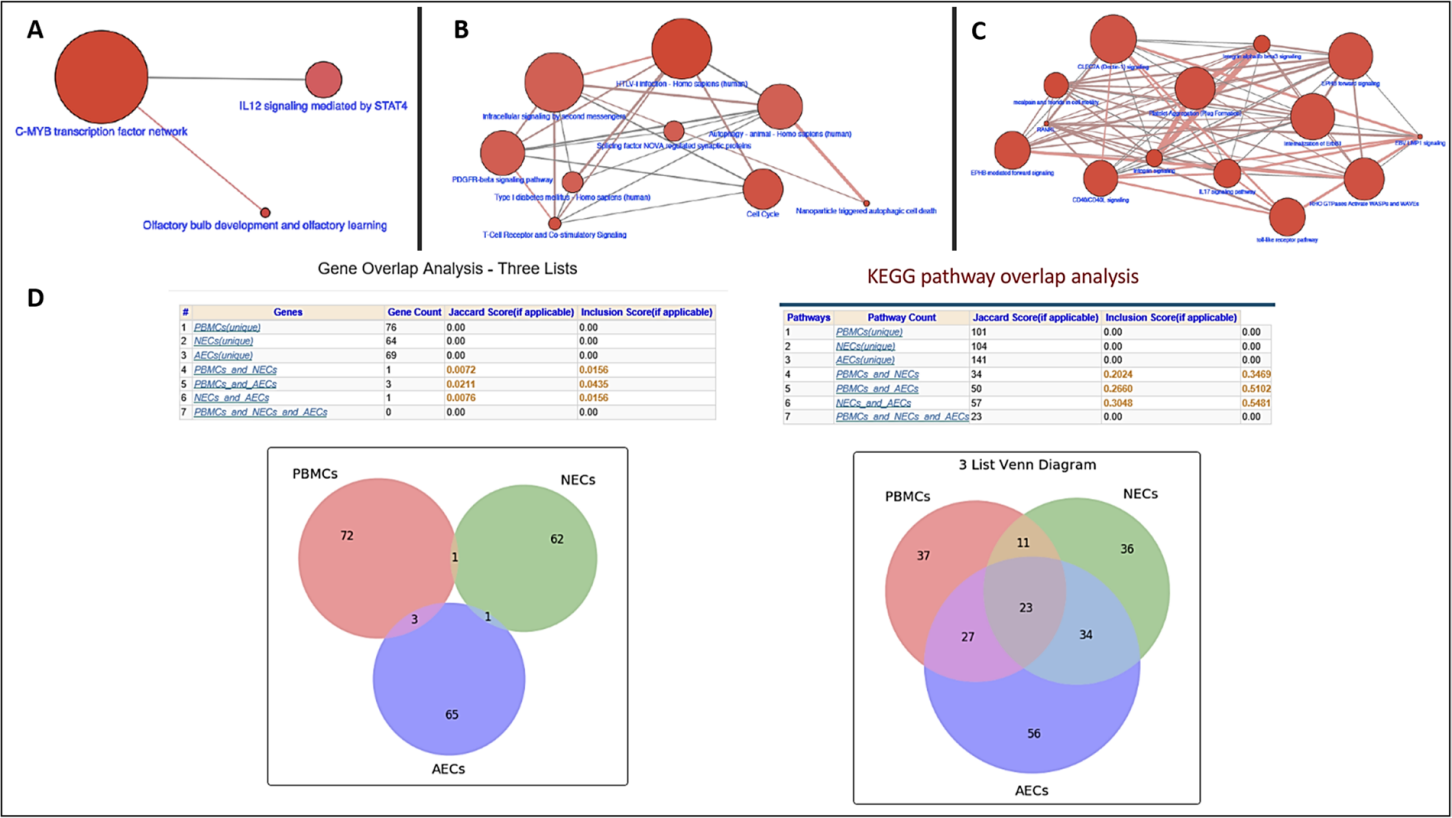
(**A**) The hub pathways for genetic networks associated with asthma based on differential trait-associated DNAm signatures derived from PBMCs. (**B**) The hub pathways for genetic networks associated with asthma based on differential trait-associated DNAm signatures derived from NECs. (**C**) The hub pathways for genetic networks associated with asthma based on differential trait-associated DNAm signatures derived from AECs. (**D**) Overlapped candidate genes and pathways between PBMC-, NEC- and AEC-based methylation data are shown.

Table 1 presents the summary of each dataset including the GEO accession number, tissue type, sample sizes, and reference to the original publication. In total, we had DNA methylation data on AECs (74 asthmatic and 41 controls), NECs (15 asthmatic and 14 controls) and PBMCs (97 asthmatic and 97 controls) samples. In order to compare the prediction errors across AEC, NEC and PBMC datasets, we used the top 100 ranked significant probes based on the single-locus model from each dataset. Figure 2A–C, show the cluster accuracy analysis results for top 100 asthma-related loci from each dataset. Both AEC and the NEC data set yielded lower two-cluster classification errors than the PBMC data set (chi-square test of independence p-value = 0.00023). The two-cluster classification errors were not statistically significantly different between the AEC and NEC datasets. Figure 3A–C, reveal the gene over-representation analysis results from the top 100 asthma-related loci from each dataset. The genes that harbored the top 100 loci in either AEC or NEC data set seemed to be over-represented in more immune-regulation-related pathways than the PBMC data set. Additionally, the AEC-based gene list also yielded more over-represented pathways than NEC-based gene list.

### Overlapped candidate genes across three different tissues

To answer the question of how often the same set of probes/genes is selected from different sample sources, we studied the overlap pattern of the top 100 genes selected from different cells/tissues. Using the top 100 ranked probes/genes from each cell/tissue, we only identified one overlapped gene from the three gene sets derived from the DNA samples extracted from the PBMCs (76 genes), NECs (64 genes), and AECs (69 genes), respectively. However, we identified 101, 104, and 141 asthma-associated genetic pathways based on the genes derived from the PBMC, NEC, and AEC data, respectively. Among these three sets of pathways derived from three different tissues, 23 pathways were shared. To compare the degree of similarity between the DNAm levels at the same probes from various tissues, we used the Jaccard similarity index. The Jaccard index scores, based on KEGG pathway overlap analysis, for the PBMCs-NECs, PBMCs-AECs, and NECs-AECs, were 0.20, 0.27, and 0.30, respectively (Fig. 3D). The overlap in differential gene methylation between tissues was observed at the gene level to a lower degree than at the pathway/functional level. The highest (J = 0.30) and lowest (J = 0.20) Jaccard similarity index among tissues/cells were for pathways between AECs and NECs and PBMCs and NECs, respectively. Since NECs are readily accessible from patients and controls, we suggest using it as model system for asthma DNA methylation studies.

### Prediction accuracy of candidate biomarkers

**Figure 4:**
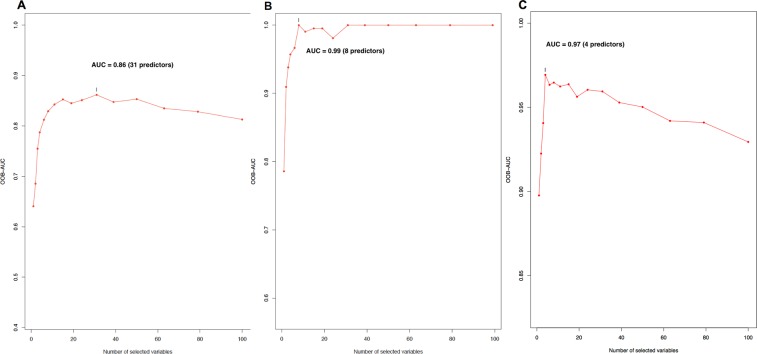
Diagnostic accuracy and efficiency levels reflected by the area under the out-of-bag curve based on DNA methylation levels from PBMCs (panel A), NECs (panel B), and AECs (panel C).

We believe that DNAm patterns of multiple loci, at least for those with larger marginal effects, exert joint effects on the diagnosis. To further explore how the DNAm levels predict the diagnosis of asthma in the context of multi-locus effects, we used Random Forest classification algorithm (RF) to identify the optimal subset of probes from the tissue-specific top-ranked 100 probes for each dataset. Each optimal subset of predictors could be used to compute the prediction errors. The area-under-curve (AUC) of prediction errors in the absence of the association between the subsets of probes and the outcome (i.e., out-of-bag or OOB errors) could then be used to infer the corresponding diagnostic ability. Figure 4A–C, show that AUC based on OOB errors was 0.88, 0.99, and 0.97 for the PBMC, NEC, and AEC datasets, respectively. Additionally, the numbers of loci in the optimal set of predictors that corresponded with the largest OOB error, were 31, 8, and 4 for the PBMC, NEC, and AEC datasets, respectively. The results suggest that both the AEC-based and NEC-based DNAm levels might predict the diagnosis of asthma more accurately (i.e., higher prediction accuracy based on the OOB-based AUC levels). In addition, both NEC-based and AEC-based DNAm data required a smaller set of probes (i.e., fewer than 10) to achieve the maximum prediction accuracy than PBMC-based DNAm data – which required 31 probes to achieve the maximum prediction accuracy. Therefore, both NEC-based and AEC-based DNAm data exerted a better diagnostic efficiency than the PBMC-based DNAm data.

### Feature selection for predictor importance

**Figure 5:**
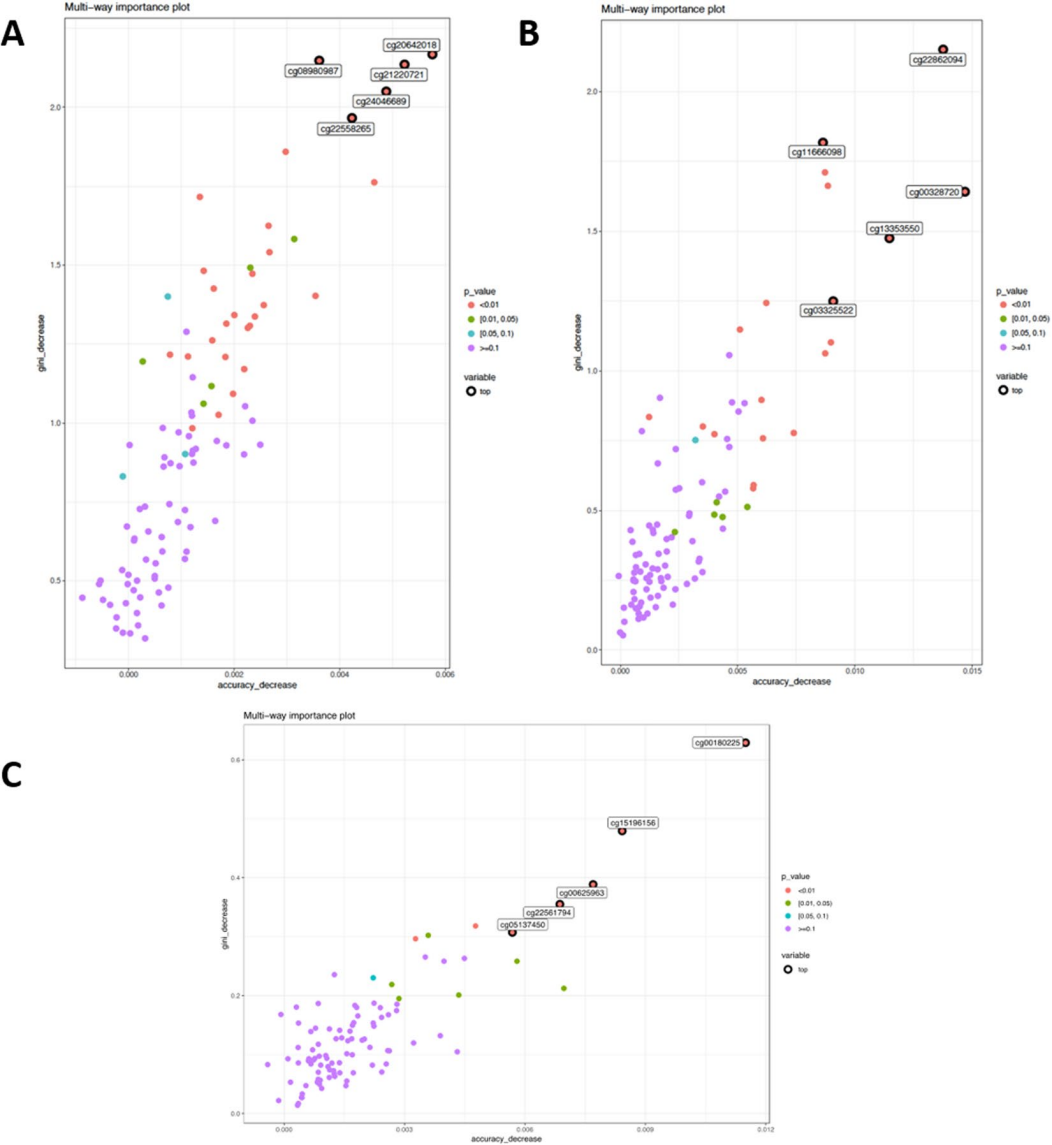
Multi-way importance levels of top asthma associated loci with DNAm data derived from three different tissues are shown (panel A: PBMCs, panel B: NECs, panel C: AECs). Gini decrease value indicates the decrease in the Gini index. Accuracy decrease indicates the prediction error based on OOB errors in the absence of the target predictor (CpG site).

Rank-based feature selection is one of the frequently used criteria in many feature selection methods that apply one or more ranking scores to separate the highly relevant features from the least relevant features. Here, we used the combination of two RF indexes (i.e., decrease in the Gini index and decrease in prediction accuracy) to rank the relative importance of the predictors that included probes and demographic factors and surrogate variables. Again, RF-based feature selection could allow us to identify top predictors in the context of the multi-locus model. The p-values were computed using the one-sided binomial test to denote the probability of split on the predictor as if it was uniformly drawn from all candidate variables for PBMC-based DNAm (Supplementary Table 1), NEC-based DNAm (Supplementary Tables 2), and AEC-based DNAm (Supplementary Table 3). Figure 5A–C, show the importance of differential methylation regions based on these two indexes for prediction performance based on PBMC, NEC, and AEC data, respectively. The three optimal sets of predictors shared no CpG site. Note that NEC dataset had relatively lower Gini index values for the five most important loci (range: 0.3–0.7) compared to the AEC or PBMC dataset – which might be partially attributable to it relatively smaller sample size.

### Locus-locus interactions

**Figure 6:**
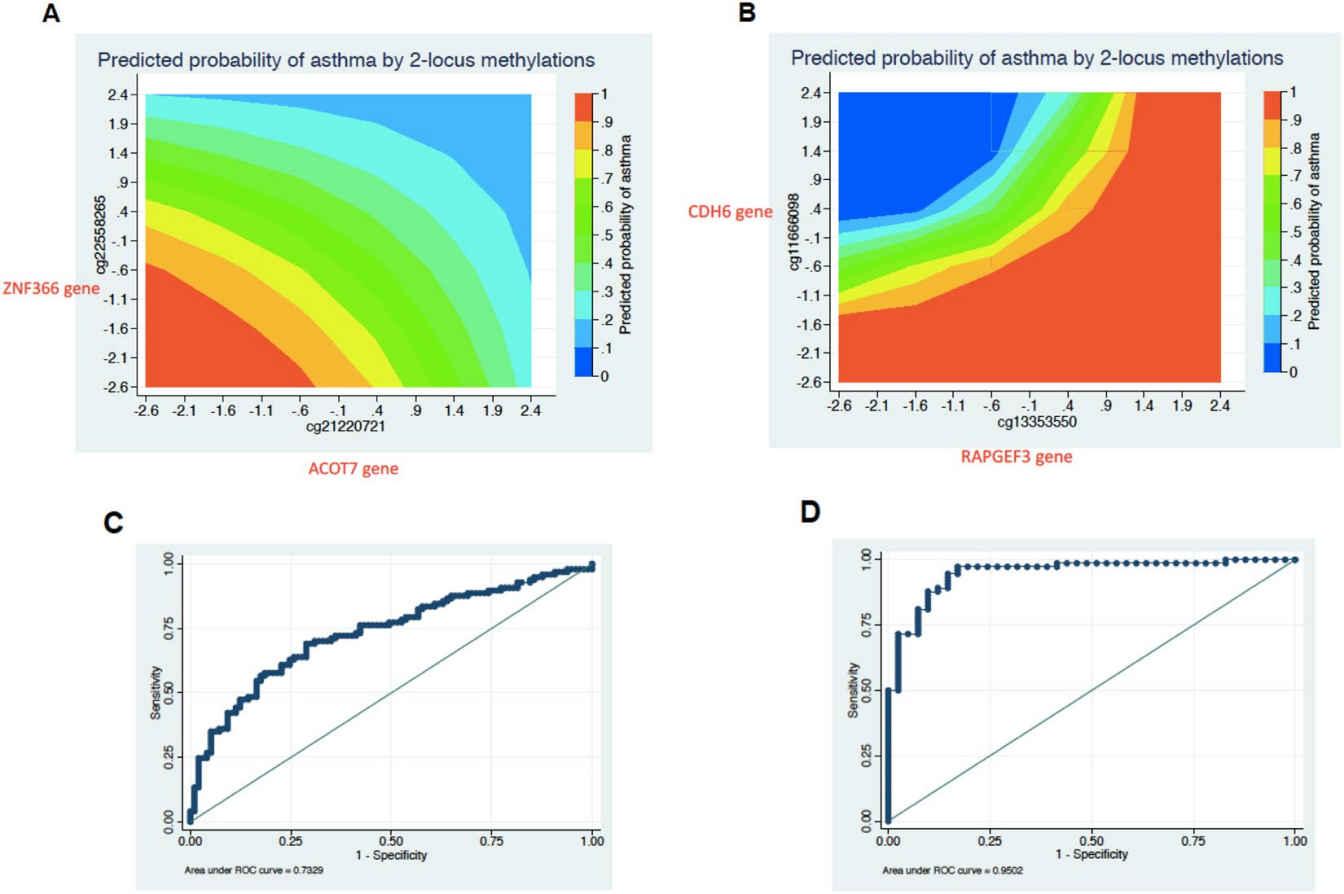
Locus-locus interactions are presented: (**A**) Heat map of the risk of asthma indicated by two loci’s methylation levels based on the PBMC data, (**B**) Heat map of the risk of asthma indicated by two loci’s methylation levels based on AECs data, (**C**) The ROC curve predicted by the PBMC-based locus-locus interaction model is shown, and (**D**) The ROC curve predicted by the AECs-based locus-locus interaction model is shown.

The locus-locus interaction analysis is an essential method to evaluate how epigenetic modifications of multiple loci might have a non-linear relationship to jointly influence the risk of asthma. The evidence for locus-locus DNAm might indicate the presence of long-range control of gene transcription. Supplementary Fig. 1 shows how the risk of asthma might depend on different combinations of DNAm levels across two loci. The most probable pair of loci with methylation patterns that interacted with each other from the PBMC data comprised cg22558265 reside in ZNF366 gene and cg21220721 located in the ACOT7 gene (Supplementary Fig. S1A). The greatest risk of asthma occurred when both loci were hypo-methylated (see Fig. 6A). The most probable pair of loci with methylation patterns that interacted with each other from the AEC data comprised two CpG sites including cg11666098 in the CDH6 gene (Supplementary Fig. S1A) and cg13353550 (in the RAPGEF3 gene). Hypomethylated conditions of the cg13353550 increased the risk of asthma by decreasing methylation levels of the cg11666098; however, if cg13353550 is hypermethylated the risk of asthma might no longer depend on the methylation levels of cg11666098 (see Fig. 6B). No statistical two-locus interactions were detected in the NEC data’s optimal set of predictors. The ROC curves of the best two-locus interaction and associated AUC values of the PBMC- and AEC-based models were 0.73 (Fig. 6C) and 0.91 (Fig. 6D), respectively.

## Discussion

In spite of numerous DNA methylation studies of asthma using samples from various types of tissues, several questions are not systematically addressed. For example, how often are the same sets of genes selected from different tissues/cells? To what degree do gene lists based on different tissues/cells share common sets of pathways? How do gene lists based on different tissues/cells perform in predicting the diagnosis of asthma? The current study aims to provide some clues to these questions. It is, hence, important to understand how the selection of sources of DNA samples might impact the identification of molecular targets associated with asthma.

Cellular heterogeneity may contribute to the differences in DNAm profile among tissues/cells. To further evaluate whether the selection of the optimal subset of CpG sites was confounded by cellular heterogeneity, we used the reference-based method proposed by Zheng and colleagues^40^ to determine the proportion of different types of cells between cases and controls. The results suggest that the proportion of immune cells relative to epithelial cells or fibroblasts was not statistically significantly different between cases and controls in either NEC or AEC data (Supplementary Fig. S2A,B). The RF analysis that was used to determine the optimal subset of CpG sites suggests that the list of top five CpG sites remained the same after adjusting for cellular heterogeneity (Supplementary Fig. S3). Similarly, we did not find the difference in the proportion of eosinophils in the blood samples between cases and controls. These results indicate that confounding effect, if present, may not be a big problem when our major goal is prediction^41^.

Our findings suggest that NECs may provide comparable diagnostic performances with AECs, both of which have better diagnostic performance than PBMCs. The PBMC data show that cytosolic acyl coenzyme A thioester hydrolase 7 (ACOT7) gene might act in concert with another candidate gene, Zinc Finger Protein 366 (ZNF366) gene, to jointly influence the risk of asthma. The role of methylations of ACOT7 gene in asthma has been reported by several genome-wide epigenomic studies on serum immunoglobin E (IgE) levels^42–44^. One of the top five loci based on the NEC data, cg00625963, is located in the LCK Proto-Oncogene, Src Family Tyrosine Kinase (LCK) gene, which can mediate the allergic airway inflammation in asthma^45–47^. The AEC data show that Rap Guanine Nucleotide Exchange Factor 3 (RAPGEF3) gene might act in concert with another candidate gene, cadherin-6 (CDH6) gene, to jointly influence the risk of asthma. The expression of the RAPGEF3 gene (also known as EPAC gene) has been found to play a role in neutrophil dysfunction^48^ and airway smooth muscle remodeling^49^. Both genes have been found to be involved in cell-cell junction, which may play a pivotal role in the pathophysiology of asthma. The interaction between DNAm levels of these two genes seem to lend some support to this mechanism.

The candidate genes from these three different tissues share a significant proportion of pathways linked to asthma. For example, the MAPK-signaling pathway and IL17 signaling pathway is involved in the pathological processes of allergic diseases^50^. The asthma-associated genes derived from PBMCs and AECs have yielded completely different “hub” pathways. The candidate genes derived from the PBMC-based methylation profiles were enriched in only three pathways, primarily in the C-MYB transcription factor network. On the contrary, the candidate genes derived from the AEC-based samples were enriched in thirteen pathways, many of which are related to immune regulation, such as IL17 signaling pathway, CD40/CD40L signaling, Dectin-1 signaling, and receptor activator of nuclear factor kappa-Β ligand pathway (RANKL, also known as tumor necrosis factor ligand superfamily member 11). The role of IL17 receptor gene in asthma has been reported by several studies^51–54^. Additionally, the AEC-based candidate genes are also enriched in two integrin-related pathways, which play a role in host cell defense systems. Furthermore, AEC-based genes are also enriched in two pathways: Platelet Aggregation and Rho GTPases pathway. Rho-related C3 botulinum toxin substrate 1 in the platelet is involved in the airway smooth muscle proliferation, which is a key component in the pathophysiology of asthma^55^. Our DNAm analysis shows that the overlap is more remarkable at the pathway level than the gene level across three tissues – which suggests that asthma involves differentially regulated biological pathways rather than individual genes in isolation.

The current study has several limitations. First, AEC-based DNAm data came from an adult population, while the other two datasets came from pediatric populations. The age-dependent DNAm levels might lead to biased associations. In our current study, we have adjusted for the age effect in each tissue-specific EWAS, and hence the top 100 probes from each EWAS were associated with asthma regardless of the age variation in each tissue-specific dataset. Note that NEC-based and AEC-based sets of optimal subsets of predictors were found to yield similar diagnostic performance. Previous evidence has shown that NECs and AECs have similar responses to cytokine stimulation and comparable expression of relevant surface receptors^56^. Therefore, the two sets of markers of tissue-specific DNAm levels might predict asthma with comparable accuracy and efficiency despite the age difference. Second, the NECs came from a relatively smaller sample size compared with PBMCs or AECs. Therefore, the NEC-based data analysis results warrant replications with a larger sample to validate the current findings. Additionally, this might have led to an insufficient statistical power to detect locus-locus interactions. Third, we did not control for medication effects, which might have an impact on epigenetic patterns and hence might confound the results. Fourth, the variable timings of DNA extraction might contribute to the variation in methylations. The present study has notable strengths. We determined whether readily accessible tissue could be used as a reliable surrogate marker to predict DNA methylation in less accessible tissues, which would facilitate the development of novel differential methylation-based models for assessing asthma risk and progression. Our data suggest that both AEC- and NEC-based DNAm data had better predictive accuracy (i.e., lower classification errors) and efficacy (i.e., require fewer loci in the optimal set of predictors) levels than the PBMC-based DNAm data. Furthermore, we obtained sets of the tissue-specific candidate loci from EWAS, which might have generated some false positive results in spite of the adjustment of confounders, especially heterogeneity in cellular composition. However, the purpose of our study is not to definitively identify genes that influence the risk of asthma through epigenetic modifications. Instead, we aimed to evaluate DNAm markers as diagnostic biomarkers. Therefore, it may not be necessary to remove the effects of all confounders. Notably, the ranks of the top five probes with DNAm levels associated with the diagnosis remained the same after we adjusted for batch effect and cellular heterogeneity using the surrogate variable analysis. Finally, our study might be subject to model overfitting since we did not evaluate the prediction accuracy in independent samples. However, the purpose of our study is to compare diagnostic performance across different tissue-specific sets of probes, so the concern of model overfitting might not substantially impact the validity of the conclusions^57^.

It is important to note that our analyses, and hence interpretations, are subject to additional limitations. The study is based on DNA methylation and associated clinical outcome data available in the public domain. Due to lack of available clinical data, the number of covariates is restricted to existing data. Associations that stemmed from un-adjusted covariates or confounding factors, such as non-biologically-related experimental variations, might still be present. Nevertheless, the present study focused on the predictive value of epigenetic markers. Therefore, our findings of the optimal subset of predicting CpG sites may not be affected by unknown confounders. If un-adjusted confounders were involved in the optimal subset of predictors, they might attenuate the predictive value of these CpG sites. To clarify whether un-adjusted confounders exerted an impact on the selection of predictors, we have also compared the results of the RF analysis that incorporated one of the potential confounders, cellular heterogeneity with the results of the RF analysis without taking cellular heterogeneity into account. These findings suggest that machine learning algorithm with a focus on prediction may be less susceptible to confounders. Nevertheless, such predictors may need further verification to clarify whether they have unbiasedly predicted as well as unravel biological mechanisms underlying the disease.

## Conclusion

We have shown that methylation patterns from AECs and NECs might serve as better biomarkers for the diagnosis of asthma compared with methylation patterns derived from PBMCs. The comparable diagnostic accuracy and efficiency levels of NECs and AECs suggest that the immune-related genetic functional measurements derived from NECs might serve as biomarkers associated with pathological changes in the central airway. In addition, the comparable diagnostic accuracy between NECs and AECs suggests that NECs may be considered as practical surrogate biomarkers for the diagnostics of asthma. Our data also suggest that DNA methylation profiling based on PBMCs may not accurately reflect DNAm profiles seen in the airway. Furthermore, NEC-based or AEC-based DNAm profiles associated with asthma reflect more genetic networks centered on immune regulation, compared with PBMC-based DNA methylation signatures associated with asthma. Profiling DNAm levels in tissues/cells that directly contribute to asthma pathogenesis is likely to aid the discovery of novel drug targets and biomarkers. Therefore, caution needs to be exercised when interpreting the results from epigenetic studies using DNA extracted from PBMCs.

In conclusion, the main purpose of this study is to identify a panel of biomarkers to aid asthma diagnostics. This purpose is different from the attempt to search for genuine biological causes of the disease. The prediction using biomarkers is likely less susceptible to biases that arise from un-adjusted or unmeasured confounders, compared with studies that aim to identify etiological factors. Nevertheless, some confounders, such as certain environmental exposures, might predict the risk of asthma well. Adding such confounders might further enhance diagnostic performance of biomarkers. Future analysis should consider additional confounders to increase the predictive value of the predicting CpG sites.

## Supplementary information

Supplementary Information

## Data Availability

All data generated and analyzed in this study is available from the GEO (http://www.ncbi.nlm.nih.gov/geo/).
