# Supplementary material for: Comparing DNA methylation profiles across different tissues associated with the diagnosis of pediatric asthma
Source: Sci Rep. 2020 Jan 13;10:151. doi: 10.1038/s41598-019-56310-4 (PMC6957523; doi:10.1038/s41598-019-56310-4)
Supplement: Supplementary file 1 — Supplementary Information [file 41598_2019_56310_MOESM1_ESM.docx]

Comparing DNA methylation profiles across different tissues associated with the diagnosis of pediatric asthma

Ping-I Lin, MD, PhD^1,^ Huan Shu, PhD^1,2^, and Tesfaye B. Mersha, PhD^3*^

^1^Department of Health Sciences, Karlstad University, Karlstad, Sweden, ^2^ Department of Environmental Science and Analytical Chemistry, Stockholm University, Stockholm, Sweden,^3^Division of Asthma Research, Department of Pediatrics, Cincinnati Children’s Hospital Medical Center, University of Cincinnati, Cincinnati, OH, USA.

*Corresponding author:

Tesfaye B. Mersha, PhD, MS

Associate Professor

Division of Asthma Research

Department of Pediatrics

Cincinnati Children’s Hospital Medical Center,

3333 Burnet Avenue, Cincinnati, OH 45229-3039, USA

Phone: 513-803-2766

Fax: 513-636-1657

E-mail: [tesfaye.mersha@cchmc.org](mailto:tesfaye.mersha@cchmc.org)

Table S1. The table summarizes the importance analysis results using the Random Forest classification algorithm for top 100 probes derived from the PBMC-based DNAm data. The p-value was computed using the one-sided binomial test to denote the probability of split on the predictor as if it was uniformly drawn from all candidate variables.

| Probe | Mean of minimum depth | No. of nodes | Gini decrease | No. of trees | P-value |
| --- | --- | --- | --- | --- | --- |
| cg08980987 | 5.29777228 | 150 | 1.97709754 | 129 | 1.91E-12 |
| cg20642018 | 5.34991089 | 146 | 1.65654017 | 127 | 2.38E-11 |
| cg22558265 | 5.47530693 | 137 | 1.4175438 | 122 | 4.63E-09 |
| cg00123072 | 5.54145545 | 126 | 1.23088685 | 115 | 1.32E-06 |
| cg24046689 | 5.45050495 | 124 | 1.87454744 | 107 | 3.34E-06 |
| cg21220721 | 5.37824752 | 122 | 1.87726467 | 113 | 8.20E-06 |
| cg20038219 | 5.73052475 | 119 | 1.09503714 | 103 | 2.97E-05 |
| cg15905634 | 5.73215842 | 115 | 0.93412968 | 98 | 0.00014785 |
| cg00618291 | 5.67838614 | 114 | 1.01112758 | 105 | 0.00021632 |
| cg04983687 | 5.46056436 | 112 | 1.47549549 | 105 | 0.00045171 |
| cg10536955 | 5.63546535 | 112 | 1.34380435 | 100 | 0.00045171 |
| cg24597466 | 5.68760396 | 112 | 1.11173783 | 98 | 0.00045171 |
| cg17653190 | 5.71119802 | 111 | 0.91144824 | 97 | 0.00064459 |
| cg19357305 | 5.61945545 | 108 | 1.45742165 | 99 | 0.00178042 |
| cg09055743 | 5.80178218 | 105 | 0.85536548 | 94 | 0.00455286 |
| cg09646558 | 5.83891089 | 104 | 0.83503522 | 94 | 0.00611902 |
| cg09696385 | 5.57374257 | 104 | 1.45831254 | 98 | 0.00611902 |
| cg11682508 | 5.67291089 | 103 | 0.97369143 | 96 | 0.00815248 |
| cg14582642 | 5.71794059 | 103 | 1.08780838 | 90 | 0.00815248 |
| cg24323275 | 5.68807921 | 103 | 1.10585988 | 92 | 0.00815248 |
| cg18460809 | 5.74153465 | 101 | 1.12010596 | 89 | 0.01409544 |
| cg03716672 | 5.89631683 | 99 | 0.72883945 | 88 | 0.02352589 |
| cg20263733 | 5.6940297 | 96 | 1.09999383 | 95 | 0.04746247 |
| cg13390484 | 5.94961386 | 95 | 0.67640836 | 87 | 0.05891463 |
| cg04260584 | 5.87551485 | 94 | 0.77257029 | 85 | 0.07248184 |
| cg12105691 | 5.71067327 | 94 | 1.08867119 | 88 | 0.07248184 |
| cg14325123 | 5.71067327 | 93 | 1.07312049 | 88 | 0.08838356 |
| cg08919594 | 5.80009901 | 91 | 0.82707402 | 84 | 0.12796573 |
| cg11157208 | 5.84365347 | 89 | 0.94913268 | 81 | 0.17884948 |
| cg14486905 | 5.92022772 | 88 | 0.69965213 | 83 | 0.20868303 |
| cg02383666 | 5.96130693 | 87 | 0.68417187 | 80 | 0.24139363 |
| cg01004641 | 5.90685149 | 86 | 0.72288493 | 80 | 0.27684675 |
| cg09243591 | 5.89679208 | 84 | 0.62121637 | 82 | 0.35503305 |
| cg22719004 | 5.94018812 | 84 | 0.74856871 | 81 | 0.35503305 |
| cg24368962 | 5.83886139 | 84 | 1.03080564 | 79 | 0.35503305 |
| cg24902995 | 5.86888119 | 84 | 0.79175967 | 75 | 0.35503305 |
| cg01869058 | 5.85982178 | 83 | 0.77547603 | 80 | 0.3970928 |
| cg21575634 | 5.93934653 | 82 | 0.70438434 | 76 | 0.44056136 |
| cg23387863 | 5.95683168 | 82 | 0.54396607 | 74 | 0.44056136 |
| cg00881207 | 5.99527723 | 81 | 0.72492798 | 73 | 0.48493912 |
| cg05875239 | 5.92960396 | 81 | 0.76838429 | 74 | 0.48493912 |
| cg10330847 | 5.92960396 | 81 | 0.90456089 | 74 | 0.48493912 |
| cg24254387 | 5.90137624 | 80 | 0.74380807 | 71 | 0.52968702 |
| cg03113038 | 5.95435644 | 78 | 0.67763168 | 74 | 0.61805541 |
| cg01554231 | 5.98074257 | 76 | 0.70044775 | 69 | 0.70131891 |
| cg08886063 | 5.89047525 | 76 | 1.02952017 | 68 | 0.70131891 |
| cg18337287 | 5.9210198 | 75 | 0.81514698 | 73 | 0.73983916 |
| cg08640475 | 5.95846535 | 74 | 0.82213496 | 69 | 0.77577669 |
| cg17018980 | 5.99938614 | 74 | 0.61488859 | 68 | 0.77577669 |
| cg24271718 | 6.06869307 | 72 | 0.52812509 | 68 | 0.83888223 |
| cg16404170 | 6.02561386 | 70 | 0.58283017 | 65 | 0.88949954 |
| cg11625077 | 5.86224752 | 69 | 0.98879734 | 65 | 0.91015198 |
| cg06048750 | 6.06537624 | 67 | 0.52279169 | 63 | 0.94283513 |
| cg26102082 | 6.04657426 | 67 | 0.51136759 | 66 | 0.94283513 |
| cg16409452 | 6.13268317 | 66 | 0.47533035 | 57 | 0.95529932 |
| cg22217449 | 6.11356436 | 66 | 0.4571398 | 64 | 0.95529932 |
| cg00057667 | 6.14985149 | 64 | 0.3754145 | 59 | 0.97377546 |
| cg12756150 | 6.07543564 | 64 | 0.49927303 | 61 | 0.97377546 |
| cg18307978 | 6.09160396 | 64 | 0.41972888 | 60 | 0.97377546 |
| cg02400146 | 6.10413861 | 63 | 0.51183145 | 58 | 0.98033806 |
| cg09527123 | 6.07938614 | 63 | 0.48721818 | 58 | 0.98033806 |
| cg24188561 | 6.05926733 | 63 | 0.49892816 | 62 | 0.98033806 |
| cg12193345 | 6.09787129 | 62 | 0.42876823 | 59 | 0.98547336 |
| cg20949306 | 6.10266337 | 62 | 0.45936309 | 61 | 0.98547336 |
| cg26916609 | 6.07443564 | 62 | 0.49772525 | 58 | 0.98547336 |
| cg22176018 | 6.07559406 | 61 | 0.51812869 | 59 | 0.98942753 |
| cg27482571 | 6.03894059 | 60 | 0.60609424 | 53 | 0.99242273 |
| cg05593759 | 6.11419802 | 59 | 0.48430124 | 56 | 0.99465407 |
| cg15225688 | 6.08681188 | 59 | 0.42971443 | 58 | 0.99465407 |
| cg02480298 | 6.16138614 | 57 | 0.44883992 | 54 | 0.99746514 |
| cg03538296 | 6.13284158 | 57 | 0.39400423 | 55 | 0.99746514 |
| cg05355684 | 6.12178218 | 57 | 0.54160274 | 54 | 0.99746514 |
| cg14026109 | 6.0820198 | 57 | 0.58427719 | 56 | 0.99746514 |
| cg02304156 | 6.16665347 | 56 | 0.3357903 | 50 | 0.99829759 |
| cg09494176 | 6.2369604 | 55 | 0.35101289 | 53 | 0.99887612 |
| cg13922442 | 6.11583168 | 55 | 0.44454376 | 51 | 0.99887612 |
| cg27363280 | 6.14321782 | 55 | 0.44850605 | 49 | 0.99887612 |
| cg07035442 | 6.18513861 | 53 | 0.42162975 | 51 | 0.99953547 |
| cg07714085 | 6.22126733 | 50 | 0.30292311 | 48 | 0.99989252 |
| cg25029657 | 6.27093069 | 50 | 0.24951029 | 46 | 0.99989252 |
| cg18304186 | 6.16070297 | 49 | 0.37445913 | 47 | 0.99993652 |
| cg01616956 | 6.18561386 | 47 | 0.40524369 | 45 | 0.99997916 |
| cg07208891 | 6.1100396 | 47 | 0.57264902 | 46 | 0.99997916 |
| cg22521333 | 6.22174257 | 47 | 0.25935441 | 42 | 0.99997916 |
| cg15161691 | 6.24006931 | 46 | 0.30380602 | 45 | 0.99998842 |
| cg22069247 | 6.15591089 | 46 | 0.41963435 | 45 | 0.99998842 |
| cg25470384 | 6.26977228 | 46 | 0.2703648 | 45 | 0.99998842 |
| cg03068030 | 6.26482178 | 45 | 0.29068613 | 45 | 0.99999371 |
| cg09505513 | 6.27372277 | 45 | 0.27461008 | 42 | 0.99999371 |
| cg10917426 | 6.21168317 | 45 | 0.31832742 | 44 | 0.99999371 |
| cg09788778 | 6.19451485 | 43 | 0.30412322 | 42 | 0.99999826 |
| cg06636203 | 6.25292079 | 42 | 0.22141867 | 39 | 0.99999912 |
| cg13775629 | 6.28262376 | 41 | 0.25671181 | 39 | 0.99999956 |
| cg14525703 | 6.25555446 | 40 | 0.3309596 | 37 | 0.99999979 |
| cg16412914 | 6.23559406 | 40 | 0.38394254 | 39 | 0.99999979 |
| cg09875326 | 6.32486139 | 39 | 0.21958284 | 37 | 0.9999999 |
| cg18151262 | 6.28409901 | 39 | 0.23624015 | 36 | 0.9999999 |
| cg00805874 | 6.28294059 | 35 | 0.24603967 | 35 | 1 |
| cg06967316 | 6.3589901 | 35 | 0.16285549 | 28 | 1 |
| cg02407068 | 6.35914851 | 26 | 0.1547233 | 26 | 1 |

.

Table S2. The table summarizes the importance analysis results using the Random Forest classification algorithm for top 100 probes derived from the NEC-based DNAm data. The p-value was computed using the one-sided binomial test to denote the probability of split on the predictor as if it was uniformly drawn from all candidate variables.

| Probe | Mean of minimum depth | No. of nodes | Gini decrease | No. of trees | P-value |
| --- | --- | --- | --- | --- | --- |
| cg13353550 | 3.28386383 | 88 | 1.43180273 | 81 | 7.49E-16 |
| cg11666098 | 3.34308936 | 77 | 1.39619353 | 72 | 2.61E-11 |
| cg22862094 | 3.31842553 | 74 | 1.52256117 | 70 | 3.47E-10 |
| cg13570892 | 3.3597617 | 68 | 1.59129678 | 63 | 4.26E-08 |
| cg01062020 | 3.5972766 | 58 | 0.49818803 | 55 | 4.00E-05 |
| cg23913941 | 3.49089362 | 57 | 0.99081162 | 55 | 7.27E-05 |
| cg00328720 | 3.49645106 | 53 | 1.36965075 | 52 | 0.00066983 |
| cg13089729 | 3.57730213 | 53 | 0.58826487 | 52 | 0.00066983 |
| cg10600644 | 3.57860426 | 51 | 0.67178649 | 49 | 0.00183113 |
| cg03325522 | 3.60074894 | 50 | 0.71299625 | 47 | 0.002947 |
| cg27404351 | 3.71651064 | 48 | 0.55307757 | 45 | 0.00722381 |
| cg18773597 | 3.66162553 | 47 | 0.73849113 | 44 | 0.01099754 |
| cg20417128 | 3.68203404 | 47 | 0.50778416 | 46 | 0.01099754 |
| cg22992279 | 3.72415319 | 47 | 0.34935675 | 47 | 0.01099754 |
| cg10166277 | 3.67525957 | 46 | 0.50193506 | 42 | 0.0164282 |
| cg05514883 | 3.6999234 | 44 | 0.43637504 | 44 | 0.03460379 |
| cg13237021 | 3.66205957 | 44 | 0.61055328 | 43 | 0.03460379 |
| cg03875819 | 3.60335319 | 42 | 0.97267704 | 41 | 0.06741163 |
| cg24707200 | 3.65441702 | 42 | 0.97265341 | 41 | 0.06741163 |
| cg07138070 | 3.75315745 | 40 | 0.55678039 | 39 | 0.12132432 |
| cg20872072 | 3.6084766 | 39 | 0.9871752 | 39 | 0.15796838 |
| cg23088126 | 3.79188936 | 39 | 0.43647342 | 38 | 0.15796838 |
| cg01298102 | 3.83018723 | 38 | 0.42611436 | 38 | 0.2016189 |
| cg10371155 | 3.76635745 | 38 | 0.71715179 | 38 | 0.2016189 |
| cg18198301 | 3.75402553 | 38 | 0.37806761 | 37 | 0.2016189 |
| cg09203501 | 3.78806809 | 37 | 0.38926259 | 37 | 0.25226684 |
| cg14329285 | 3.69914043 | 37 | 0.87850338 | 36 | 0.25226684 |
| cg20595453 | 3.88724255 | 37 | 0.24745822 | 34 | 0.25226684 |
| cg27659109 | 3.68637447 | 37 | 0.94380499 | 36 | 0.25226684 |
| cg07670266 | 3.76765957 | 36 | 0.35350889 | 35 | 0.30946994 |
| cg11733958 | 3.65658723 | 36 | 1.12757809 | 36 | 0.30946994 |
| cg13355449 | 3.77148085 | 36 | 0.34208794 | 36 | 0.30946994 |
| cg00146864 | 3.85702128 | 35 | 0.16059672 | 35 | 0.37231131 |
| cg00579868 | 3.73874043 | 35 | 0.76384955 | 33 | 0.37231131 |
| cg08579420 | 3.78554894 | 35 | 0.68502301 | 33 | 0.37231131 |
| cg20290361 | 3.79788085 | 34 | 0.36874977 | 34 | 0.43940856 |
| cg20702417 | 3.73874043 | 34 | 0.50866708 | 33 | 0.43940856 |
| cg20729768 | 3.71702979 | 34 | 0.75672866 | 34 | 0.43940856 |
| cg00415333 | 3.83279149 | 33 | 0.34029969 | 32 | 0.5089818 |
| cg13881964 | 3.86214468 | 33 | 0.20872411 | 33 | 0.5089818 |
| cg15033552 | 3.82471489 | 32 | 0.29709272 | 31 | 0.57897927 |
| cg19396253 | 3.7689617 | 32 | 0.74443991 | 32 | 0.57897927 |
| cg03003434 | 3.82558298 | 30 | 0.45093207 | 29 | 0.71173226 |
| cg17164345 | 3.88941277 | 30 | 0.35619516 | 29 | 0.71173226 |
| cg19670610 | 3.8166383 | 30 | 0.72165111 | 30 | 0.71173226 |
| cg24663521 | 3.85580426 | 30 | 0.18922529 | 28 | 0.71173226 |
| cg07611933 | 3.77495319 | 29 | 0.64673349 | 28 | 0.77065726 |
| cg07754940 | 3.85580426 | 29 | 0.28376389 | 28 | 0.77065726 |
| cg11702866 | 3.89922553 | 28 | 0.23788153 | 26 | 0.82269212 |
| cg11895596 | 3.86431489 | 28 | 0.38329803 | 28 | 0.82269212 |
| cg24310959 | 3.88645957 | 28 | 0.39829535 | 26 | 0.82269212 |
| cg07243202 | 3.92901277 | 27 | 0.38074011 | 26 | 0.86704452 |
| cg14394451 | 3.85198298 | 27 | 0.31593333 | 27 | 0.86704452 |
| cg03301498 | 3.92180426 | 26 | 0.23979012 | 23 | 0.90348745 |
| cg18195165 | 3.89071489 | 26 | 0.32816747 | 26 | 0.90348745 |
| cg21643265 | 3.91668085 | 26 | 0.21374834 | 25 | 0.90348745 |
| cg21735516 | 3.86605106 | 26 | 0.4622935 | 24 | 0.90348745 |
| cg11303839 | 3.90434894 | 25 | 0.30902815 | 24 | 0.93231362 |
| cg03002688 | 3.88732766 | 24 | 0.16476525 | 24 | 0.95423132 |
| cg06516476 | 3.95115745 | 24 | 0.1040521 | 24 | 0.95423132 |
| cg09666573 | 3.89158298 | 24 | 0.40814147 | 24 | 0.95423132 |
| cg15321306 | 3.90434894 | 24 | 0.23634997 | 24 | 0.95423132 |
| cg18052713 | 3.92562553 | 24 | 0.16852748 | 24 | 0.95423132 |
| cg09075968 | 3.93500426 | 23 | 0.18597883 | 22 | 0.97022479 |
| cg24611351 | 3.8877617 | 23 | 0.18154734 | 23 | 0.97022479 |
| cg01476222 | 3.89714043 | 21 | 0.31619391 | 21 | 0.9888799 |
| cg02532341 | 3.9354383 | 21 | 0.26061539 | 21 | 0.9888799 |
| cg02920600 | 3.93118298 | 21 | 0.33313185 | 21 | 0.9888799 |
| cg05011468 | 3.95245957 | 21 | 0.13095778 | 21 | 0.9888799 |
| cg19811148 | 3.90182979 | 21 | 0.43417698 | 20 | 0.9888799 |
| cg24436462 | 3.91459574 | 21 | 0.21794648 | 20 | 0.9888799 |
| cg21385480 | 3.95714894 | 20 | 0.24730206 | 20 | 0.99364782 |
| cg23186952 | 3.91034043 | 20 | 0.32378812 | 20 | 0.99364782 |
| cg26542856 | 3.96652766 | 20 | 0.12914315 | 18 | 0.99364782 |
| cg02660643 | 3.92822979 | 19 | 0.28677387 | 18 | 0.99654361 |
| cg04427254 | 3.96609362 | 19 | 0.27827881 | 19 | 0.99654361 |
| cg05856951 | 3.94907234 | 19 | 0.20836181 | 19 | 0.99654361 |
| cg06898168 | 3.91077447 | 19 | 0.11854206 | 19 | 0.99654361 |
| cg15835664 | 3.88142128 | 18 | 0.48752894 | 18 | 0.99821392 |
| cg21164975 | 3.94525106 | 18 | 0.15889162 | 18 | 0.99821392 |
| cg21556967 | 3.94525106 | 18 | 0.25765435 | 18 | 0.99821392 |
| cg01352294 | 3.99674894 | 17 | 0.16930468 | 17 | 0.99912638 |
| cg01541846 | 3.94994043 | 17 | 0.26760692 | 17 | 0.99912638 |
| cg03618113 | 3.93717447 | 17 | 0.25914282 | 17 | 0.99912638 |
| cg24016995 | 3.98398298 | 17 | 0.08815031 | 17 | 0.99912638 |
| cg24152264 | 3.95419574 | 17 | 0.19575522 | 17 | 0.99912638 |
| cg24178897 | 3.9669617 | 17 | 0.14938997 | 17 | 0.99912638 |
| cg05469819 | 3.98441702 | 16 | 0.12353941 | 16 | 0.99959701 |
| cg16407069 | 3.98867234 | 16 | 0.08989685 | 16 | 0.99959701 |
| cg14118475 | 3.98102979 | 15 | 0.1444158 | 14 | 0.9998254 |
| cg25701646 | 4.02358298 | 15 | 0.08705752 | 14 | 0.9998254 |
| cg03896685 | 4.00699574 | 14 | 0.17247671 | 13 | 0.99992927 |
| cg11798406 | 3.98954043 | 14 | 0.20016631 | 14 | 0.99992927 |
| cg17654419 | 4.02914043 | 12 | 0.07726372 | 11 | 0.99999072 |
| cg26025505 | 4.03339574 | 12 | 0.0613435 | 11 | 0.99999072 |
| cg03318469 | 4.04702979 | 11 | 0.05716092 | 9 | 0.99999703 |
| cg17758048 | 4.02575319 | 11 | 0.11619281 | 9 | 0.99999703 |
| cg22760287 | 4.02914043 | 11 | 0.05948007 | 11 | 0.99999703 |
| cg16395183 | 4.05979574 | 9 | 0.05004754 | 9 | 0.99999977 |
| cg22540135 | 4.05979574 | 9 | 0.03065521 | 9 | 0.99999977 |

Table S3. The table summarizes the importance analysis results using the Random Forest classification algorithm for top 100 probes derived from the AEC-based DNAm data. The p-value was computed using the one-sided binomial test to denote the probability of split on the predictor as if it was uniformly drawn from all candidate variables.

| Probe | Mean of minimum depth | No. of nodes | Gini decrease | No. of trees | P-value |
| --- | --- | --- | --- | --- | --- |
| cg22862094 | 3.07705263 | 85 | 2.22656831 | 84 | 1.08E-14 |
| cg13353550 | 3.29571053 | 78 | 1.33578908 | 77 | 8.03E-12 |
| cg23913941 | 3.36192983 | 68 | 1.36545199 | 68 | 3.40E-08 |
| cg11666098 | 3.50602632 | 63 | 1.05774026 | 61 | 1.30E-06 |
| cg10600644 | 3.6215614 | 56 | 0.69824432 | 50 | 0.00011139 |
| cg13089729 | 3.63803509 | 55 | 0.46156096 | 52 | 0.00019693 |
| cg00328720 | 3.4620614 | 54 | 1.07629833 | 53 | 0.00034225 |
| cg05514883 | 3.62102632 | 53 | 0.49914473 | 51 | 0.00058455 |
| cg10371155 | 3.47960526 | 53 | 1.25962998 | 53 | 0.00058455 |
| cg13570892 | 3.48014035 | 53 | 1.25548867 | 52 | 0.00058455 |
| cg10166277 | 3.67087719 | 51 | 0.59583185 | 48 | 0.00161704 |
| cg20417128 | 3.63140351 | 51 | 0.65378473 | 48 | 0.00161704 |
| cg13355449 | 3.73880702 | 48 | 0.52773822 | 44 | 0.00649219 |
| cg18773597 | 3.65547368 | 47 | 0.67316751 | 44 | 0.00994101 |
| cg03325522 | 3.53598246 | 46 | 1.12007053 | 46 | 0.01493539 |
| cg09203501 | 3.66371053 | 46 | 0.57690952 | 45 | 0.01493539 |
| cg11733958 | 3.57984211 | 46 | 1.24832777 | 46 | 0.01493539 |
| cg18198301 | 3.67740351 | 46 | 0.54335788 | 44 | 0.01493539 |
| cg24707200 | 3.54528947 | 46 | 1.17883968 | 45 | 0.01493539 |
| cg01062020 | 3.77496491 | 45 | 0.38040811 | 42 | 0.02201189 |
| cg08579420 | 3.64392105 | 44 | 0.98773911 | 41 | 0.0318176 |
| cg21735516 | 3.69708772 | 43 | 0.69537152 | 40 | 0.04509908 |
| cg03002688 | 3.71901754 | 42 | 0.65138043 | 40 | 0.06267418 |
| cg01298102 | 3.82920175 | 40 | 0.39128364 | 39 | 0.11401834 |
| cg07138070 | 3.68831579 | 40 | 0.89413648 | 40 | 0.11401834 |
| cg20872072 | 3.67676316 | 39 | 0.94715049 | 37 | 0.1492348 |
| cg24663521 | 3.81379825 | 39 | 0.30336089 | 35 | 0.1492348 |
| cg27404351 | 3.79026316 | 39 | 0.35285719 | 38 | 0.1492348 |
| cg20702417 | 3.77325439 | 38 | 0.53830072 | 37 | 0.19144954 |
| cg14329285 | 3.73977193 | 37 | 0.79636775 | 34 | 0.24074075 |
| cg19396253 | 3.84064912 | 37 | 0.31878835 | 34 | 0.24074075 |
| cg27659109 | 3.72500877 | 37 | 0.80894715 | 37 | 0.24074075 |
| cg07670266 | 3.7567807 | 36 | 0.38206603 | 35 | 0.29676302 |
| cg14394451 | 3.82695614 | 36 | 0.28992042 | 35 | 0.29676302 |
| cg15033552 | 3.78748246 | 36 | 0.30623672 | 35 | 0.29676302 |
| cg23088126 | 3.79133333 | 36 | 0.42769388 | 36 | 0.29676302 |
| cg13237021 | 3.82471053 | 35 | 0.37919639 | 31 | 0.35869582 |
| cg22992279 | 3.85434211 | 35 | 0.26219966 | 33 | 0.35869582 |
| cg13881964 | 3.82802632 | 33 | 0.28525228 | 33 | 0.49467695 |
| cg00146864 | 3.91242983 | 32 | 0.15245915 | 31 | 0.56497806 |
| cg02920600 | 3.84717544 | 31 | 0.31423644 | 30 | 0.63397689 |
| cg03875819 | 3.81155263 | 31 | 0.49942586 | 31 | 0.63397689 |
| cg09666573 | 3.82524561 | 31 | 0.48332741 | 30 | 0.63397689 |
| cg20595453 | 3.91403509 | 30 | 0.19865925 | 28 | 0.69956139 |
| cg00579868 | 3.77753509 | 29 | 0.72318776 | 29 | 0.75987127 |
| cg02532341 | 3.83455263 | 29 | 0.38981617 | 29 | 0.75987127 |
| cg24310959 | 3.89702632 | 29 | 0.44318011 | 27 | 0.75987127 |
| cg02660643 | 3.89371053 | 27 | 0.350333 | 25 | 0.85943651 |
| cg03003434 | 3.88878947 | 27 | 0.39625853 | 26 | 0.85943651 |
| cg06898168 | 3.91457018 | 27 | 0.1591107 | 27 | 0.85943651 |
| cg21385480 | 3.91564035 | 27 | 0.16761593 | 25 | 0.85943651 |
| cg20729768 | 3.85370175 | 26 | 0.50031662 | 26 | 0.89744806 |
| cg00415333 | 3.92879825 | 25 | 0.22205807 | 25 | 0.92770521 |
| cg05011468 | 3.93318421 | 25 | 0.15509294 | 25 | 0.92770521 |
| cg18195165 | 3.82845614 | 25 | 0.47279887 | 24 | 0.92770521 |
| cg20290361 | 3.86739474 | 25 | 0.32265132 | 25 | 0.92770521 |
| cg24152264 | 3.88055263 | 25 | 0.24017895 | 25 | 0.92770521 |
| cg26542856 | 3.92109649 | 25 | 0.25556719 | 23 | 0.92770521 |
| cg01541846 | 3.91178947 | 24 | 0.33095132 | 24 | 0.95085651 |
| cg03301498 | 3.87231579 | 24 | 0.48099974 | 24 | 0.95085651 |
| cg07754940 | 3.89424561 | 24 | 0.29875231 | 24 | 0.95085651 |
| cg07611933 | 3.91671053 | 23 | 0.36510647 | 23 | 0.96785704 |
| cg21556967 | 3.91285965 | 23 | 0.16862318 | 22 | 0.96785704 |
| cg21643265 | 3.96110526 | 23 | 0.17588962 | 22 | 0.96785704 |
| cg24611351 | 3.9825 | 23 | 0.13069394 | 23 | 0.96785704 |
| cg05856951 | 3.97041228 | 22 | 0.19701627 | 21 | 0.97981714 |
| cg15321306 | 3.89970175 | 22 | 0.36487083 | 22 | 0.97981714 |
| cg21164975 | 3.95725439 | 22 | 0.12681344 | 21 | 0.97981714 |
| cg11303839 | 3.92270175 | 21 | 0.28972574 | 20 | 0.98786295 |
| cg03318469 | 3.9895614 | 19 | 0.08368899 | 18 | 0.99618478 |
| cg05469819 | 3.95447368 | 19 | 0.21251517 | 18 | 0.99618478 |
| cg16407069 | 3.98078947 | 19 | 0.09002225 | 18 | 0.99618478 |
| cg17164345 | 4.01095614 | 19 | 0.13166433 | 19 | 0.99618478 |
| cg24436462 | 3.93639474 | 19 | 0.2098554 | 19 | 0.99618478 |
| cg25701646 | 4.00218421 | 19 | 0.0803736 | 19 | 0.99618478 |
| cg09075968 | 3.9895614 | 18 | 0.16405215 | 18 | 0.99801719 |
| cg11895596 | 3.95885965 | 18 | 0.28669883 | 18 | 0.99801719 |
| cg18052713 | 3.97255263 | 18 | 0.22047749 | 17 | 0.99801719 |
| cg19811148 | 3.99394737 | 18 | 0.2192106 | 18 | 0.99801719 |
| cg22540135 | 3.95447368 | 18 | 0.1422954 | 18 | 0.99801719 |
| cg01476222 | 4.00764035 | 17 | 0.17343133 | 17 | 0.99902455 |
| cg03618113 | 3.95939474 | 17 | 0.42376635 | 17 | 0.99902455 |
| cg11702866 | 3.95992983 | 17 | 0.21101343 | 16 | 0.99902455 |
| cg24016995 | 3.95939474 | 17 | 0.15568037 | 17 | 0.99902455 |
| cg24178897 | 4.0257193 | 17 | 0.12737756 | 16 | 0.99902455 |
| cg04427254 | 3.97747368 | 16 | 0.18537951 | 16 | 0.99954741 |
| cg06516476 | 3.9999386 | 16 | 0.13600771 | 15 | 0.99954741 |
| cg17654419 | 4.03887719 | 16 | 0.09928217 | 16 | 0.99954741 |
| cg19670610 | 3.98185965 | 16 | 0.1641408 | 16 | 0.99954741 |
| cg07243202 | 4.00432456 | 15 | 0.13059069 | 15 | 0.99980276 |
| cg23186952 | 3.97800877 | 15 | 0.14843902 | 15 | 0.99980276 |
| cg03896685 | 3.97469298 | 13 | 0.33093045 | 13 | 0.99996954 |
| cg11798406 | 4.0097807 | 13 | 0.22094165 | 13 | 0.99996954 |
| cg01352294 | 3.99330702 | 12 | 0.16753206 | 11 | 0.99998933 |
| cg15835664 | 4.00154386 | 12 | 0.24480228 | 12 | 0.99998933 |
| cg17758048 | 4.0459386 | 12 | 0.10427482 | 11 | 0.99998933 |
| cg14118475 | 4.02454386 | 10 | 0.18876924 | 10 | 0.999999 |
| cg16395183 | 4.04647368 | 10 | 0.0865315 | 10 | 0.999999 |
| cg26025505 | 4.02069298 | 10 | 0.10674366 | 9 | 0.999999 |
| cg22760287 | 4.07332456 | 9 | 0.03062645 | 9 | 0.99999974 |

Figure S1. The risks of asthma that might depend on different combinations of DNAm levels across two loci are shown. The most probable pair of loci with methylation patterns that interacted with each other from the PBMC data comprised cg22558265 reside in ZNF366 gene and cg21220721 located in the ACOT7 gene (supplementary Figure S1A). The greatest risk of asthma occurred when both loci were hypo-methylated (see Figure 6A). The most probable pair of loci with methylation patterns that interacted with each other from the AEC data comprised two CpG sites including cg11666098 in the CDH6 gene (supplementary Figure S1A) and cg13353550 (in the RAPGEF3 gene).


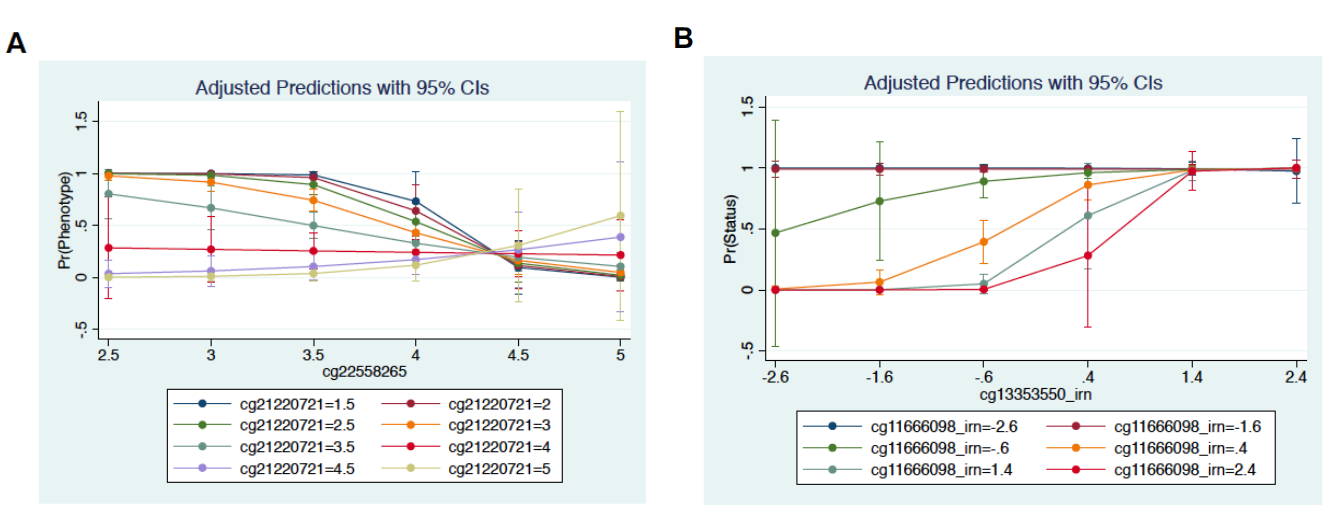


Supplementary Figure S2. The cellular composition of AEC data is shown. Epi, Fib, and IC, refer to epithelial cells, fibroblasts, and immune cells, respectively.

Supplementary Figure S3. The multi-way importance of top 100 CpG sites from the AEC data based on the RF analysis results is shown.
